# Responding to the cuts in UK AID to neglected tropical diseases control programmes in Africa

**DOI:** 10.1093/trstmh/trac109

**Published:** 2022-11-23

**Authors:** Roy M Anderson, Jorge Cano, T Déirdre Hollingsworth, Kebede Deribe-Kassaye, Honorat G M Zouré, Amir B Kello, Benido Impouma, Akpaka A Kalu, Laura Appleby, Elodie Yard, Mihretab Salasibew, Kevin McRae-McKee, Carolin Vegvari

**Affiliations:** Department of Infectious Disease Epidemiology, School of Public Health, Imperial College London, London, UK; MRC Centre for Global Infectious Disease Analysis, School of Public Health, Imperial College London, London, UK; London Centre for Neglected Tropical Disease Research, London, UK; Oriole Global Health, London, UK; Expanded Special Project for Elimination of NTDs, World Health Organization Regional Office for Africa, Brazzaville, Republic of the Congo; Big Data Institute, Li Ka Shing Centre for Health Information and Discovery, University of Oxford, Oxford, UK; Children's Investment Fund Foundation, London, UK; Expanded Special Project for Elimination of NTDs, World Health Organization Regional Office for Africa, Brazzaville, Republic of the Congo; Expanded Special Project for Elimination of NTDs, World Health Organization Regional Office for Africa, Brazzaville, Republic of the Congo; Communicable and Non-communicable Disease Cluster, World Health Organization Regional Office for Africa, Brazzaville, Republic of the Congo; Tropical and vector-borne diseases, World Health Organization Regional Office for Africa, Brazzaville, Republic of the Congo; Oriole Global Health, London, UK; Oriole Global Health, London, UK; Children's Investment Fund Foundation, London, UK; Oriole Global Health, London, UK; Department of Infectious Disease Epidemiology, School of Public Health, Imperial College London, London, UK; MRC Centre for Global Infectious Disease Analysis, School of Public Health, Imperial College London, London, UK; London Centre for Neglected Tropical Disease Research, London, UK; Oriole Global Health, London, UK

## Abstract

The early termination of the Accelerating the Sustainable Control and Elimination of Neglected Tropical Diseases (Ascend) programme by the UK government in June 2021 was a bitter blow to countries in East and West Africa where no alternative source of funding existed. Here we assess the potential impact the cuts may have had if alternative funding had not been made available by new development partners and outline new strategies developed by affected countries to mitigate current and future disruptions to neglected tropical disease control programmes.

The Accelerate the Sustainable Control and Elimination of Neglected Tropical Diseases (Ascend) programme was funded by the UK Foreign, Commonwealth and Development Office (FCDO) supporting neglected tropical disease (NTD) programmes in 23 countries in Africa and two in South Asia. The early termination of Ascend by the UK government was announced in June 2021 following a £4 billion cut to the UK's foreign aid budget in response to a fiscal emergency caused by the coronavirus disease 2019 (COVID-19) pandemic. The end of the FCDO flagship health programme to protect millions of people from NTDs was a blow to countries in sub-Saharan Africa where no alternative funding existed. Of the £220 million allocated to Ascend, £99.7 million had been spent before programme termination. Moreover, support for nutrition services was cut by 80% and support for sexual health and family planning was cut by 85%.^1,2^

NTDs affect >1 billion of the world's poorest people.^3^ Ascend aimed to support national governments in sustainably tackling NTDs, with a focus on five NTDs: trachoma, schistosomiasis (SCH), onchocerciasis, lymphatic filariasis (LF) and visceral leishmaniasis. Effective programmes for the first four of these NTDs require multiple years of uninterrupted mass drug administration (MDA) targeting at-risk populations. The aim of Ascend was to support NTD control and transfer ownership and capacity for NTD control to national governments.

Ascend had a strong integration and health system strengthening agenda that will lose traction following the discontinuation of dedicated funding. Ascend cuts indirectly affected healthcare for neglected populations because of common delivery infrastructure. For example, loss of funding for supply chains and community health workers means drugs cannot be delivered, applications for drugs are not granted and some remote communities lose access to health services.^4^ In many countries, technical personnel coordinating and managing NTD control programmes are partly funded via aid. Funding cuts lead to the loss of skills and capacity, which can take a long time to restore. The administrative units within countries that benefitted from Ascend are presented in Figure 1a. The funding gaps created by Ascend cuts have been summarized by the World Health Organization's (WHO) Expanded Special Project for Elimination of Neglected Tropical Diseases.^5^

**Figure 1.**
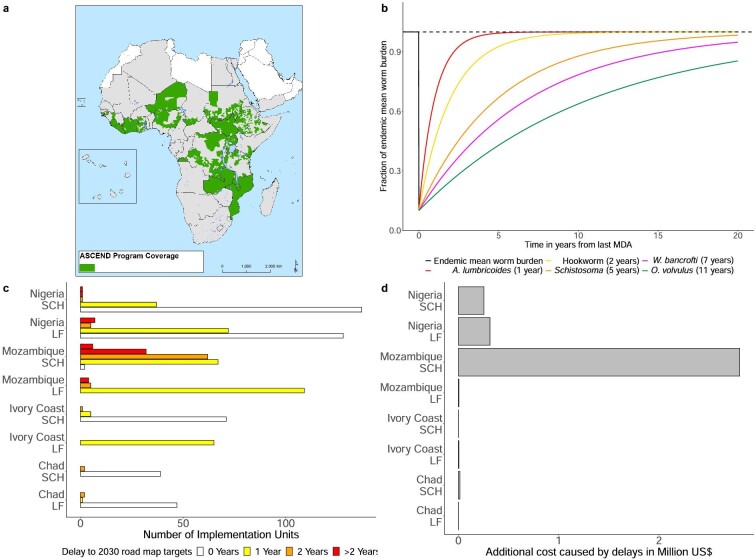
Impact of interruptions caused by cuts in UK AID on the control of NTDs via the Ascend project in combination of the concomitant impact of the COVID-19 pandemic. **(a)** Map showing MDA implementation units (IUs) in Africa that were supported by Ascend. **(b)** The schematic shows bounce-back curves of different helminth parasite species after an intense MDA round that lowers the mean equilibrium to 1/10 of its untreated value. The time to bounce-back to the baseline mean worm load per person prior to treatment after treatment is stopped depends predominantly on the mean life expectancy of the adult parasite recorded in brackets after the parasite species.^6^**(c)** Number of implementation units that would have experienced delays in reaching the targets of the WHO 2030 roadmap for LF and SCH in the least- and most-affected countries previously supported by Ascend if emergency funding had not been found. Delays depend on prevalence prior to interruptions, how many MDA rounds were missed and programme stage. **(d)** Additional costs of missed rounds due to funding cessation in the least- and most-affected countries previously supported by Ascend if emergency funding had not been found.

Assessing the impact of the funding cuts is complicated by the effects of the COVID-19 pandemic, which has delayed NTD MDA in many countries. The impact of COVID-19 has recently been assessed by the NTD Modelling Consortium.^6^

Missed MDA rounds due to funding cuts or COVID-19 interruptions can lead to a resurgence of infections called bounce-back. Time to bounce-back is disease specific and depends on the life expectancy of the adult parasite^7^ and transmission intensity. Consequently, disruptions may affect some NTDs more than others.^6^ An illustration of bounce-back times is provided in Figure 1b. For short-lived adult helminths, such as schistosomes, bounce-back occurs faster than for long-lived parasites such as filarial worms.

Figure 1c shows the number of administrative units within different countries that would experience a delay of 0, 1 or 2 y in reaching targets for SCH and LF established in the WHO 2030 roadmap for NTDs without emergency funding. Estimated additional costs of delays, in terms of additional treatments needed due to parasite bounce-back, are presented in Figure 1d. These costs are greater in countries with larger populations and for SCH (faster bounce-back) compared with the longer-lived filarial worms (slower bounce-back) (details in Supplementary Information SI1).

Programmes affected by Ascend termination resorted to adaptations in two ways. First, programmes sought alternative funding in an environment of increased pressure caused by the severe acute respiratory syndrome coronavirus 2 (SARS-CoV-2) pandemic.^8^ The donors who pledged $100 million in emergency funding over the next 2.5 y are the Children's Investment Fund Foundation, the Bill and Melinda Gates Foundation and the ELMA Foundation. Where possible, and with the agreement of ministries of health, funders sought to apply innovative methods for programme implementation and evaluation to improve efficiency and evidence gathering.

Still, not all of the funding gap has been filled. One important gap is LF morbidity management and disability prevention. Emergency funds have been made available for other major gaps. Some control programmes have restarted. Funding shortfalls for longer-term gaps left by UK AID withdrawal will remain for the foreseeable future. Funding by donors who provided emergency aid will be renewed for a subset of countries. However, emergency funding for many Ascend countries will stop in September 2022. For example, Mozambique, Tanzania and Uganda still need to fill funding gaps in LF, SCH and trachoma programmes. We undertook a gap analysis through a consultative process with the ministries of health and a clear understanding of needs (Supplementary Information SI3). Where MDA is affected, similar but delayed health and cost consequences (1–2 y gaps) are expected as for the original analysis of Ascend funding cuts (figures in Supplementary Information SI1).

The second adaptation to Ascend cuts is an initiative in the WHO African Region to enhance analytics-informed precision targeting of disease control, including for NTDs, to improve value. Precision targeting of NTD interventions will be integrated into community-based disease control as an important step towards country ownership of NTD control programmes and universal healthcare. Integration will be supported through institutional capacity strengthening for disease control research, analytics and technical support. A regional consultation on community involvement in disease control, determinants of diseases, demand creation for health services and accountability by local health stewards is planned for the second half of 2022.

UK AID for NTD control has been provided since 2008 and increased following the London Declaration on Neglected Tropical Diseases in January 2012.^9^ The cessation of Ascend during the COVID-19 pandemic was ill-timed, given the added stresses imposed by SARS-CoV-2 on health systems. Because of COVID-19 disruptions and supply chain–related factors, countries struggled to administer 88 million already delivered drugs close to expiration.^10,11^ Discontinuation of NTD control programmes resulted in missed opportunities for leveraging funding and experience from NTD MDA for COVID-19 vaccination roll-out.^12^ Ongoing NTD control programmes benefited from synergies between infection control measures for SARS-CoV-2 and NTDs.^12^

Given the strains of the COVID-19 pandemic on the economies of most countries, enhanced domestic financing should not be expected in the near term. The World Bank estimates that additional funding required for COVID-19 prevention, treatment and surveillance in sub-Saharan African countries costs 3% of gross domestic product. Simultaneously, the economies of these countries are contracting by 6%.^13^ Achieving more with available resources is therefore necessary to achieve the elimination of NTDs.^3^

## Supplementary Material

trac109_Supplemental_Files

## Data Availability

The data used for the analysis can be downloaded from the ESPEN portal. All other data and assumptions have been included in the Supplementary Information.
