# Supplementary material for: Responding to the cuts in UK AID to neglected tropical diseases control programmes in Africa
Source: Trans R Soc Trop Med Hyg. 2022 Nov 23;117(3):237–9. doi: 10.1093/trstmh/trac109 (PMC9977241; doi:10.1093/trstmh/trac109)
Supplement: trac109_Supplemental_Files [file trac109_supplemental_files.zip › FCDOCutsSI1_Analysis Details.docx]

**Impacts of cuts in UK AID on the control of the neglected tropical diseases**

# Supplementary Information 1 – Methodology

## Background

Ascend (Accelerate the Sustainable Control and Elimination of Neglected Tropical Diseases) was a programme funded by the UK Foreign, Commonwealth and Development Office. Ascend funded NTD programmes for five diseases in 25 countries in Africa (23 countries) and South Asia (2 countries). The focus in Africa was on NTDs amenable to control by preventive chemotherapy (PC). In June 2021 funding ceased earlier than originally planned, leaving many countries with a financing gap for NTD programmes, in particular PC distribution. Without new funders stepping in, this gap could have led to a delay in or even missed rounds of PC treatment that could potentially have impacted the long-term outcome of affected NTD programmes.

In 2020, the NTD Modelling Consortium showed that interruptions to NTD programmes caused by the COVID-19 pandemic can lead to delays in reaching the WHO 2030 road map targets for NTDs.^1–3^ As the cause of the interruption does not matter to the outcome, we can assume that interruptions caused by funding cuts can lead to similar delays in reaching NTD targets as interruptions caused by the pandemic. PC treatment is implemented by geographic/administrative units known as implementation units (IUs). We estimated how many implementation units in formerly Ascend supported countries were at risk of experiencing delays to reaching the WHO 2030 road map targets for lymphatic filariasis (LF) and schistosomiasis.

## Linking ESPEN with Ascend data

Data on IUs in Ascend workplans were linked with the corresponding records in the ESPEN database (Expanded Special Project for Elimination of Neglected Tropical Diseases).^4^ ESPEN records include information for each IU on treatment rounds prior to Ascend implementation, endemicity, prevalence category (for schistosomiasis, not for LF), and forecasted years for impact assessment (schistosomiasis) and transmission assessment surveys (TAS for LF). Ascend workplan data includes information on PC rounds conducted as part of the project. Linking the two datasets can be done via IU names, but this is not always straightforward because IU names may change, and IUs may be merged or split. Linking current IUs with historic IUs required close collaboration between Ascend data teams and ESPEN (WHO-AFRO).

## Lymphatic filariasis – assumptions, delay estimates and cost of delays

The WHO 2030 road map target for LF is elimination as a public health problem (EPHP), defined as <1% microfilaria prevalence. (Microfilaria are the larvae of the parasitic worms causing LF that can be detected in blood spots from infected patients.) WHO recommends annual communitywide PC treatment with at least 65% coverage for at least four to six years to reach this target.^5^ ESPEN forecasts the year in which this target will be achieved based on past treatment rounds with effective coverage. In their analysis on the impact of COVID-19 related interruptions to LF programmes, the NTD Modelling Consortium estimated the expected delay in reaching the target based on microfilaria prevalence prior to interruptions and the duration of interruptions (the gap between the last treatment round and the subsequent round).^3,6^ The expected delays for LF endemic settings in Africa, where *Anopheles* mosquitoes are the predominant vector species, are summarised in Table S1. For this analysis we only consider interruptions of one or two complete years, as the exact date of PC implementation was not available for each IU, so half-year delays could not be determined.

We rounded the estimated delays to reaching EPHP caused by interruptions to PC programmes. This means we assumed that an interruption to PC delivery of one year causes a delay in reaching EPHP of one year (EPHP would be measured one year later than planned).

Table S1: Expected delays to reaching EPHP for LF given microfilaria (mf) prevalence prior to interruptions and duration of interruption (gap between PC treatment rounds). Figures show rounded mean values of estimates by the NTD Modelling Consortium ^6^.

| Gap between PC rounds | Low prevalence  (5-10% mf) | Moderate prevalence (15-20% mf) | High prevalence  (25-30% mf) |
| --- | --- | --- | --- |
| 1 year delay (2 year gap) | 1 | 1 | 1 |
| 2 year delay (3 year gap) | 2 | 2 | 2 |

Data on mf prevalence prior to interruptions for each IU were extracted from the NTD Prevalence Simulator Beta Version (<https://ntdmodelling.net/lf>). The prevalences in the NTD Prevalence Simulator were estimated from data provided by geospatial models using a methodology developed by Touloupou *et al*.^7^ For our analysis we extracted information on the prevalence category of each IU prior to interruptions. A few IUs had no prevalence estimates, and some IUs in Ascend workplans could not be matched up with IUs in the NTD Prevalence Simulator. In these instances, we assumed that the IU with missing prevalence data would have the same prevalence category as the majority of the surrounding IUs.

We applied the estimated delays in reaching EPHP for LF to the IUs, accounting for mf prevalence category and gap between PC rounds. We assumed that all IUs could resume PC delivery without further interruptions from 2022 onwards.

Two hundred and forty-two IUs experienced no gap between PC rounds (white bars in Figure S1). These are IUs for which the 2021 PC round went ahead before funding was cut, IUs that failed (pre-)TAS and had to restart MDA or IUs for which previous treatment data was not available. 319 IUs would have been affected by interruptions to PC delivery in 2021 if new funding had not been found (yellow bars in Figure S1). An additional 105 IUs would have experienced a two-year interruption to LF programmes, because of COVID-19 in 2020 and because of funding cuts in 2021 (orange bars in Figure S1). 98 IUs experienced interruptions to their LF programmes of more than two years (i.e. PC treatment for LF had not been delivered in consecutive years prior to 2020). Reaching EPHP in these IUs is expected to be delayed by more than two years (red bars in Figure S1).

The NTD Modelling Consortium suggests a number of mitigation strategies to avoid delays caused by interruptions to LF programmes.^3,6^ Since the purpose of our analysis was to quantify the potential damage caused by interruptions to NTD programmes, we did not consider mitigation strategies here.


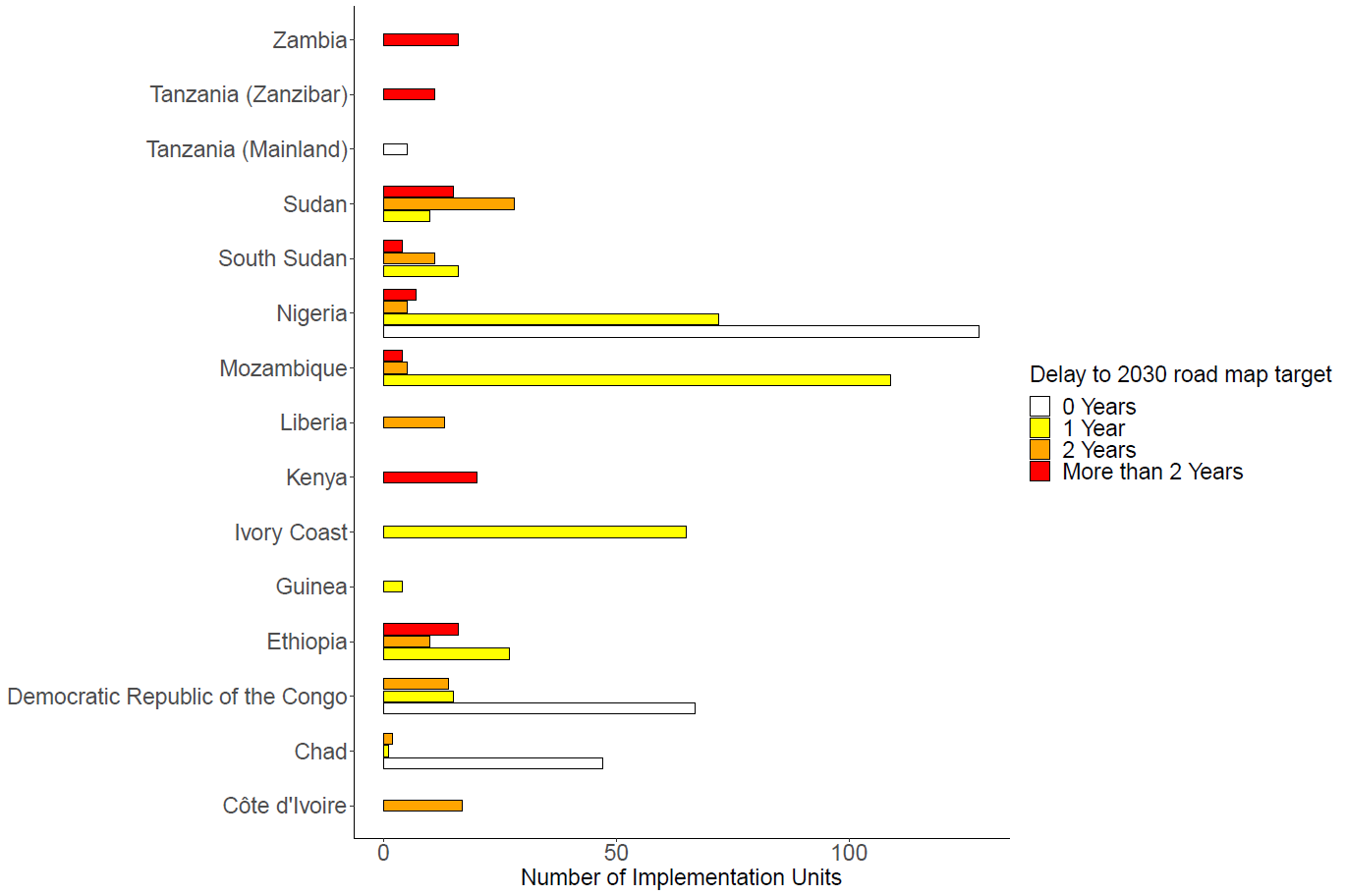


Figure S2: Number of implementation units (IUs) in the Ascend programme affected by LF programme interruptions. Only countries where Ascend supported LF programmes are shown. White bars represent IUs that did not experience LF programme interruptions. Yellow bars represent IUs that experienced a one-year interruption to LF programmes because of COVID-19 or that would have experienced a one-year interruption because of funding cuts if new funding had not been made available. Orange bars represent IUs that experienced interruptions to LF programmes caused by the COVID-19 pandemic in 2020 and would have been affected by cuts to Ascend in 2021 (two-year interruptions). Red bars represent IUs that experienced interruptions to PC for LF of more than 2 years.

Interruptions to LF programmes of one or two years mean that the missed rounds of PC treatment will have to be implemented later. Because of population growth, the delayed rounds will likely be more expensive than the 2021 round would have been. To quantify the additional cost, we used the ESPEN API to get the year when each IU was forecast to reach EPHP. We calculated the size of the population requiring PC treatment in that year using estimates of current population size and population growth rate in the ESPEN database. We then calculated the difference in population size in the year when the delayed PC treatment round would happen and the population size in the year when treatment should have happened according to plan. For each IU and delayed PC round, we multiplied the difference in the size of the population requiring treatment with the unit cost per PC treatment in the corresponding country. PC treatment unit costs were taken from Fitzpatrick *et al*. 2016.^8^ For each country, we summed the additional costs incurred by delays to reaching EPHP in each IU. The results are shown in Figure S2.


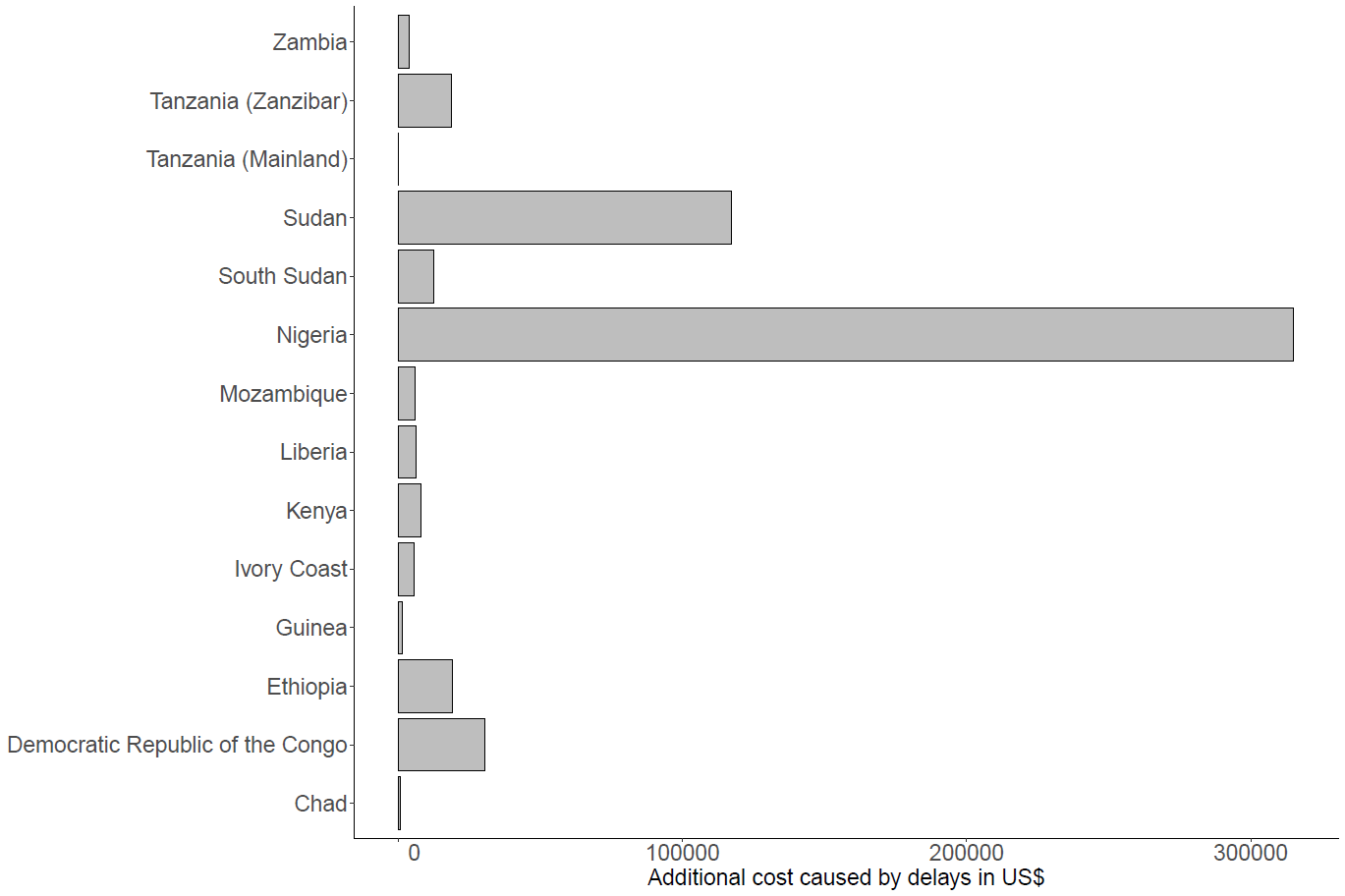


Figure S3: Additional costs to LF programmes in Ascend countries caused by delays to reaching EPHP in US$. The additional cost depends on the difference in the population size in 2021 and the later year when the round missed in 2021 will be implemented. Therefore, the magnitude of the additional cost depends on the length of the delay, the population affected by delays (population requiring treatment), population growth and the treatment cost per person.

## Schistosomiasis – assumptions, delay estimates and cost of delays

The WHO 2030 NTD road map target for schistosomiasis is elimination as a public health problem (EPHP) defined as <1% prevalence of heavy-intensity infections in school-age children (SAC). New WHO guidelines recommend annual preventive chemotherapy (PC) treatment for all age groups in implementation units with ≥10% prevalence of schistosomiasis infection (moderate-high prevalence settings). Previous WHO guidelines that were applied during the Ascend programme recommended treatment of 75% SAC and at-risk adults annually in high-prevalence settings (≥50% prevalence of schistosomiasis infection in SAC), every two years in moderate-prevalence settings (10-49% prevalence in SAC), and every three years in low-prevalence settings (<10 % prevalence in SAC).^9^ After five to six years of PC treatment, WHO recommends conducting an impact assessment survey and to reduce PC treatment frequency depending on the new prevalence or to stop PC treatment if EPHP has been reached.

In their analysis on the impact of COVID-19 related interruptions to schistosomiasis programmes, the NTD Modelling Consortium estimated the expected delay in reaching EPHP given baseline prevalence, programme stage (early or late), age-prevalence distribution and *Schistosoma* species.^3,10^ The ESPEN database includes data on schistosomiasis prevalence and number of rounds of treatment conducted, but not age-prevalence distribution or *Schistosoma* species. Therefore, we used average estimates for delays by prevalence category and programme stage as shown in Table S2.^3,10^ For this analysis we only consider interruptions of one or two complete years, as the exact date of PC implementation was not available for each IU, so half-year delays could not be determined.

Table S2: Expected delays to reaching EPHP for schistosomiasis given prevalence in SAC prior to interruptions, programme stage and duration of interruption. Figures show rounded mean values of estimates by the NTD Modelling Consortium ^3,10^.

| Gap between PC rounds | Low prevalence  (<10%) | Moderate prevalence (10-49%) | High prevalence (≥50%) |
| --- | --- | --- | --- |
| 1 year delay – early stage programme | 0 | 1 | 2 |
| 1 year delay – late stage programme | 0 | 1 | 2 |
| 2 year delay – early stage programme | 1 | 3 | 5 |
| 2 year delay – late stage programme | 1 | 2 | 3 |

We extracted the prevalence and the number of rounds of PC treatment conducted prior to interruptions from the ESPEN database for each IU. We classified IUs that had conducted up to 2 rounds of PC treatment prior to interruptions as early stage and IUs that had conducted 3 or more rounds of PC treatment prior to interruptions as late stage. The ESPEN database does not contain forecasts for when EPHP of schistosomiasis will be reached, but the time for the next impact assessment survey is available. We optimistically assume that EPHP can be reached by the time of the next impact assessment survey.

We applied the estimated delays to reaching EPHP for schistosomiasis to the IUs, accounting for prevalence category, programme stage and delay in PC rounds. As in moderate-prevalence settings PC treatment is delivered only every second year, we only assumed that an IU experienced interruptions if it was scheduled to receive PC in 2020 and/or 2021 and this treatment was not delivered. We assumed that all IUs could resume PC delivery without further interruptions from 2022 onwards.

The majority of Ascend IUs (636) will not experience delays to reaching EPHP of schistosomiasis because of programme disruptions in 2020 or 2021 (white bars Figure S3). The reason is that these IUs have low schistosomiasis prevalence or they were not scheduled to receive PC treatment when interruptions occurred. 164 IUs are expected to experience a one-year delay to reaching EPHP (yellow bars in Figure S3). 100 IUs are expected to experience a delay of two years to reaching EPHP (orange bars in Figure S3). 73 IUs are expected to experience a three-year delay to reaching EPHP (red bars in Figure S3). 19 IUs are at risk of experiencing a delay of more than three years to reaching EPHP (purple bars in Figure S3). The latter IUs have experienced interruptions to their schistosomiasis programmes of more than two years.

The NTD Modelling Consortium suggests a number of mitigation strategies to avoid delays caused by interruptions to schistosomiasis programmes.^3,10^ Since the purpose of our analysis was to quantify the potential damage caused by interruptions to NTD programmes, we did not consider mitigation strategies here.


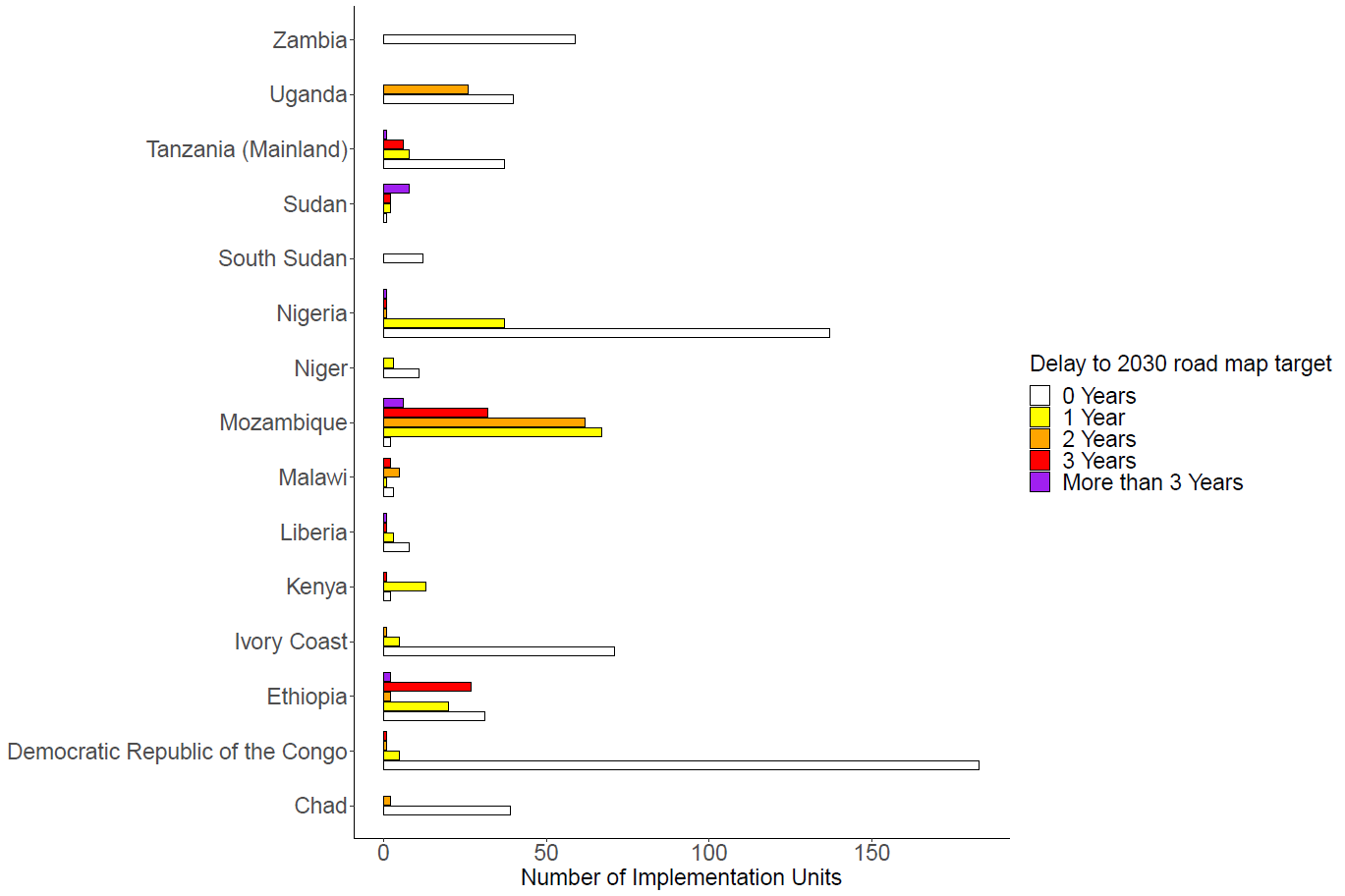


Figure S4: Number of implementation units (IUs) in the Ascend programme affected by schistosomiasis programme interruptions. Only countries where Ascend supported schistosomiasis programmes are shown. White bars represent IUs that are not expected to experience delays to EPHP of schistosomiasis because of programme interruptions in 2020 or 2021. Yellow bars represent IUs that are expected to experience a one-year delay to reaching EPHP. Orange bars represent IUs that are expected to experience a two-year delay to reaching EPHP. Red bars represent IUs that are expected to experience more than two years delay to reaching EPHP.

Interruptions to schistosomiasis programmes mean that the missed rounds need to be implemented later. Moreover, in high-prevalence settings interruptions to PC programmes for schistosomiasis the delay to EPHP is longer than the duration of the interruption. This means that additional rounds of treatment may be necessary. We calculated the additional cost caused by interruptions to schistosomiasis programmes by adding the cost of additional rounds of treatment required to reach EPHP (beyond the next scheduled impact assessment survey) plus the additional cost of delayed rounds because of population growth. Costs per treatment per person for each country were taken from Fitzpatrick *et al*. 2016.^8^ For each country, we summed the additional costs incurred by delays to reaching EPHP in each IU. The results are shown in Figure S4.


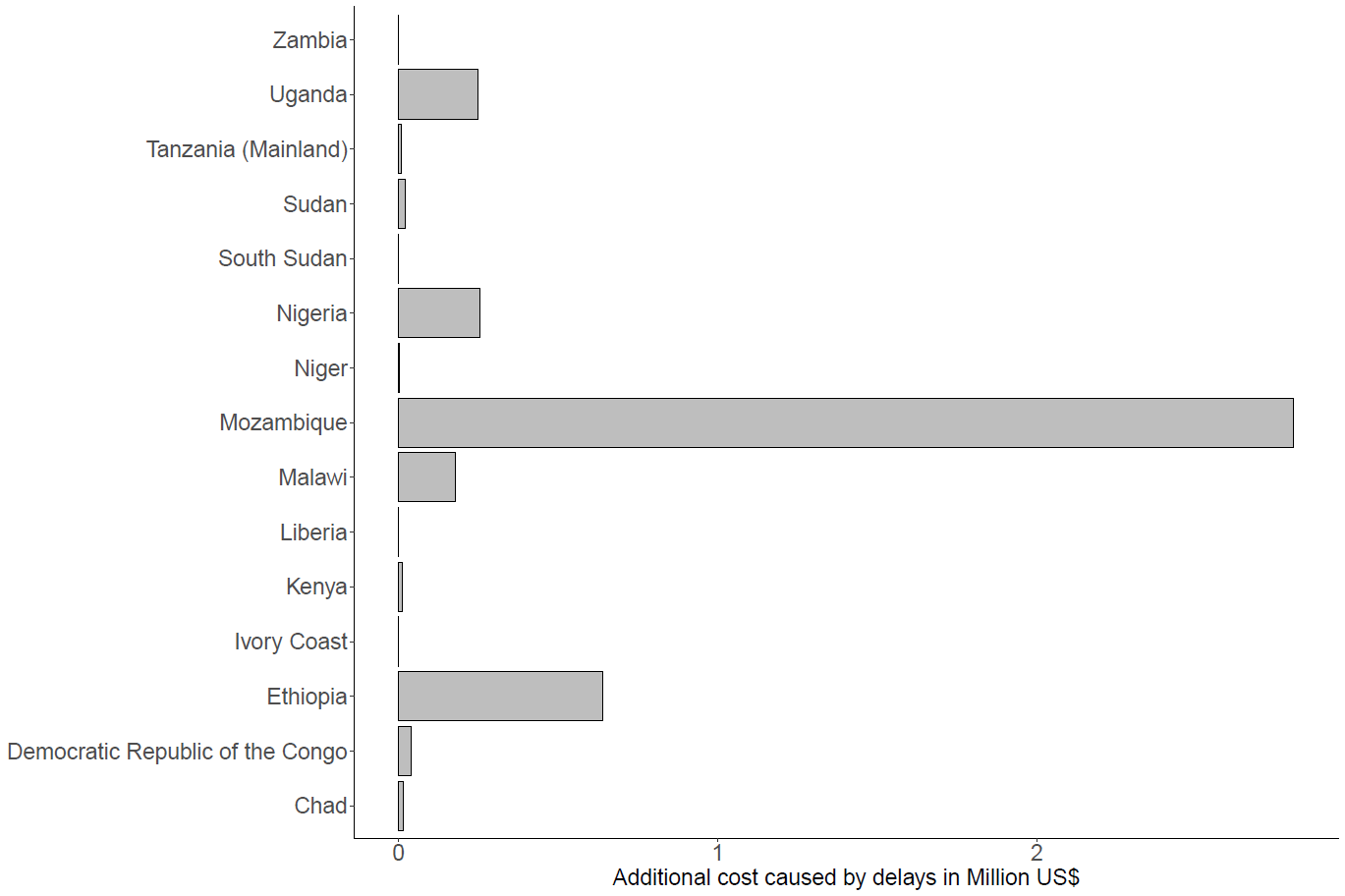


Figure S4: Additional costs to schistosomiasis programmes in Ascend countries caused by delays to reaching EPHP in million US$. The magnitude of the additional cost depends on the length of the delay, the population affected by delays (population requiring treatment), population growth (difference in population size in 2021 and in the year when delayed rounds are implemented) and the treatment cost per person.

**Supplementary Information 1 References**

1 NTD Modelling Consortium. NTD Modelling Consortium. The potential impact of programmes interruptions due to COVID-19 on 7 neglected tropical diseases: a modelling-based analysis [version 1; not peer reviewed]. *Gates Open Research*; **4**. https://doi.org/10.21955/gatesopenres.1116665.1.

2 Toor J, Hamley JID, Fronterre C, *et al.* Strengthening data collection for neglected tropical diseases: What data are needed for models to better inform tailored intervention programmes? *PLOS Neglected Tropical Diseases* 2021; **15**: e0009351.

3 World Health Organization. Impact of the COVID-19 pandemic on seven neglected tropical diseases: a model-based analysis. Geneva, Switzerland: World Health Organization, 2021.

4 World Health Organization Regional Office for Africa. Expanded Special Project For Elimination of Neglected Tropical Diseases. 2021 https://espen.afro.who.int/.

5 World Health Organization. Lymphatic filariasis - Key facts. Geneva, Switzerland, 2021 https://www.who.int/news-room/fact-sheets/detail/lymphatic-filariasis.

6 Prada JM, Stolk WA, Davis EL, *et al.* Delays in lymphatic filariasis elimination programmes due to COVID-19, and possible mitigation strategies. *Trans R Soc Trop Med Hyg* 2021; **115**: 261–8.

7 Touloupou P, Retkute R, Hollingsworth TD, Spencer SEF. Statistical methods for linking geostatistical maps and transmission models: Application to lymphatic filariasis in East Africa. *Spatial and Spatio-temporal Epidemiology* 2020; : 100391.

8 Fitzpatrick C, Fleming FM, Madin-Warburton M, *et al.* Benchmarking the Cost per Person of Mass Treatment for Selected Neglected Tropical Diseases: An Approach Based on Literature Review and Meta-regression with Web-Based Software Application. *PLoS Negl Trop Dis* 2016; **10**: e0005037–e0005037.

9 World Health Organization. Helminth control in school-age children: a guide for managers of control programmes. Geneva, Switzerland: World Health Organization, 2011.

10 Kura K, Ayabina D, Toor J, Hollingsworth TD, Anderson RM. Disruptions to schistosomiasis programmes due to COVID-19: an analysis of potential impact and mitigation strategies. *Transactions of The Royal Society of Tropical Medicine and Hygiene* 2021; **115**: 236–44.
