# Supplementary material for: Responding to the cuts in UK AID to neglected tropical diseases control programmes in Africa
Source: Trans R Soc Trop Med Hyg. 2022 Nov 23;117(3):237–9. doi: 10.1093/trstmh/trac109 (PMC9977241; doi:10.1093/trstmh/trac109)
Supplement: trac109_Supplemental_Files [file trac109_supplemental_files.zip › FCDOCutsSI2_List of Supported Countries.docx]

**Impacts of cuts in UK AID on the control of the neglected tropical diseases**

**Supplementary Information 2**

| Countries in Africa supported by Ascend: | Countries in Asia supported by Ascend: |
| --- | --- |
| Benin  Burkina Faso  Central African Republic  Chad  Côte d’Ivoire  Democratic Republic of the Congo  Ethiopia  Ghana  Guinea  Guinea-Bissau  Kenya  Liberia  Malawi  Mozambique  Niger  Nigeria  Sierra Leone  South Sudan  Sudan  Tanzania (Mainland)  Tanzania (Zanzibar)  Uganda  Zambia | Bangladesh  Nepal |
