# Supplementary material for: Responding to the cuts in UK AID to neglected tropical diseases control programmes in Africa
Source: Trans R Soc Trop Med Hyg. 2022 Nov 23;117(3):237–9. doi: 10.1093/trstmh/trac109 (PMC9977241; doi:10.1093/trstmh/trac109)
Supplement: trac109_Supplemental_Files [file trac109_supplemental_files.zip › FCDOCutsSI3_CostBreakDownRemaingGaps.docx]

**Impacts of cuts in UK AID on the control of the neglected tropical diseases**

# Supplementary Information 3 – Breakdown of remaining funding gaps in three Ascend countries for a period of 18 months (August 2022 – January 2024)

# The aim of Ascend was to support NTD control and transferring ownership and capacity for NTD programme delivery to national governments. Ascend targeted four main pathways for delivery: prevention of NTDs through MDA, clinical treatment of the debilitating effects of disease through morbidity management and disability prevention (MMDP); increasing knowledge for targeting interventions through NTD mapping, and focused monitoring, evaluation and learning activities; and skills transfer through the systems and governance pathway.

**Mozambique:**

| ***Intervention*** | ***Intervention Gap*** | | ***Cases*** | ***Target IUs*** | ***Target***  ***Population*** | ***Indicative Budget (£)*** |
| --- | --- | --- | --- | --- | --- | --- |
| Lymphatic filariasis | MMDP | Lymphoedema | 8,760 | 51 |  | 1,054,794 |
|  |  | Hydrocelectomy | 15,000 | 51 |  |  |
|  | Prevention | MDA |  | 56 | 12.3 M | 950,000 |
|  | Monitoring | TAS |  | 77 |  |  |
| SCH/STH | Prevention | MDA |  |  |  | 1,400,000 |
|  | Precision mapping | | | 77 |  | 1,400,000 |
| SBCC/WASH-NTD |  | | | | | 175,000 |
| EME |  | | | | | 100,000 |
| HSS | SCH/STH elimination strategy, capacity building, workshops and training | | | | | 250,000 |
|  | | | | | | **3,929,794** |

**Uganda:**

| ***Intervention*** | ***Intervention Gap*** | | ***Cases*** | ***Target IUs*** | ***Target Population*** | ***Indicative***  ***Budget (£)*** |
| --- | --- | --- | --- | --- | --- | --- |
| Lymphatic filariasis | MMDP | **Lymphoedema** | 7,000 | 26 | *-* | 400,000 |
|  |  | Hydrocelectomy | 8,000 |  |  |  |
| Trachoma |  | TT Surgeries | 8,000 | 20 | *-* | 800,000 |
| SCH/STH | Prevention | MDA | | 77 | 7,400.000 | 1,300,000 |
|  | Precision Mapping | | | 25 | **-** | 1,998,000 |
| SBCC/WASH-NTD |  | | | | | 175,000 |
| EME |  | | | | | 100.000 |
| HSS | SCH/STH elimination strategy and human capacity | | | | | 130,000 |
|  |  | | | | | **4,903,000** |

**Tanzania**:

| ***Intervention*** | ***Intervention Gap*** | | ***Cases*** | ***Target IUs*** | ***Target Population*** | ***Indicative***  ***Budget (£)*** |
| --- | --- | --- | --- | --- | --- | --- |
| Lymphatic filariasis | MMDP | Lymphoedema | 13,500 | **-** | 150,000 | 405,000 |
|  |  | Hydrocelectomy | 5,850 | **-** | 450,000 | 526,500 |
| Trachoma |  | TT Surgeries | 100 | 1 | - | 250,000 |
| Lymphatic filariasis | Monitoring | LF TAS |  | 15 | 123,000 | 123,000 |
|  |  | Precision mapping |  | 51 | 150,000 | 90,000 |
| SCH/STH |  | SCH Precision Mapping/Impact Assessment of SCH/STH |  | 50 | 450,000 | 450,000 |
| SCH/LF | Prevention | Test and Treat |  | - | - | 340,000 |
| SCH |  | FGS |  | 10 | 884 | 317,000 |
| SBCC, WASH-NTD |  |  |  |  |  | 350,000 |
| EME |  | | | | | 100,000 |
| HSS | SCH/STH elimination strategy, capacity building workshops and training | | | | | 228,000 |
|  |  | | | |  | **3,179,500** |
